# Assessing the structure and meaningfulness of the dissociative subtype of PTSD

**DOI:** 10.1007/s00127-017-1445-2

**Published:** 2017-09-29

**Authors:** Jana Ross, Gabriel Baník, Mária Dědová, Gabriela Mikulášková, Cherie Armour

**Affiliations:** 1 Psychology Research Institute, Ulster University, Coleraine, BT52 1SA Northern Ireland UK; 2 Faculty of Arts, Institute of Psychology, University of Prešov, Prešov, Slovakia; 3 Faculty of Arts, School of Psychology, Trnava University in Trnava, Trnava, Slovakia

**Keywords:** PTSD, Dissociation, Subtype, DSM-5, Latent profile analysis

## Abstract

**Purpose:**

Studies conducted in the USA, Canada and Denmark have supported the existence of the dissociative PTSD subtype, characterized primarily by symptoms of depersonalization and derealization. The current study aimed to examine the dissociative PTSD subtype in an Eastern European, predominantly female (83.16%) sample, using an extended set of dissociative symptoms.

**Methods:**

A latent profile analysis was applied to the PTSD and dissociation data from 689 trauma-exposed university students from Slovakia.

**Results:**

Four latent profiles of varying PTSD and dissociation symptomatology were uncovered. They were named non-symptomatic, moderate PTSD, high PTSD and dissociative PTSD. The dissociative PTSD profile showed elevations on depersonalization and derealization, but also the alternative dissociative indicators of gaps in awareness and memory, sensory misperceptions and cognitive and behavioural re-experiencing. The core PTSD symptoms of ‘memory impairment’ and ‘reckless or self-destructive behaviour’ were also significantly elevated in the dissociative PTSD profile. Moreover, anxiety and anger predicted membership in the dissociative PTSD profile.

**Conclusion:**

The results provide support for the proposal that the dissociative PTSD subtype can be characterized by a variety of dissociative symptoms.

## Introduction

The relationship between trauma, PTSD and dissociation has been well documented in the literature [1, 2] and a number of different models have been proposed to account for this relationship [3]. Perhaps, the most prominent of these models that has garnered a lot of attention in recent years is the Subtype model of dissociative PTSD. Recognizing the importance of this model, the DSM-5 [4] introduced within its nosology a diagnostic category of a dissociative subtype of PTSD. To qualify for this diagnosis, trauma survivors have to meet the full criteria for PTSD and additionally report experiences of depersonalization and/or derealization, characterized by ‘out-of-body’ experiences and ‘feelings of unreality’ respectively. The aim of the current study was to examine the support for the Subtype model of the relationship between PTSD and dissociation.

Dalenberg, Glaser and Alhassoon [5] argued that before a relationship between two variables can be considered a disorder subtype, (1) there needs to be a clear definition of the construct, (2) the disorder and its subtype must have a differential structure and/or be fuelled by differential biological mechanisms and (3) the existence of the disorder subtype must be meaningful. With respect to the first criterion, the DSM-5 has defined the dissociative PTSD subtype in terms of depersonalization and derealization symptoms and a number of studies have identified these symptoms in individuals with PTSD [6]. With respect to the differential structure criterion, studies have shown that the dissociative and non-dissociative PTSD do differ in their basic structure (i.e. severity of specific PTSD symptoms). In recent years, statistical techniques of latent class (LCA) and latent profile analysis (LPA) have become popular in comparing the structure of dissociative and non-dissociative PTSD. LCA/LPA categorizes individuals into different classes/profiles based on their endorsements or severity of individual symptoms. Since there are currently 20 different PTSD symptoms in DSM-5, which allows for the diagnosis of PTSD to be given in 636,120 different ways [7], LCA and LPA are the ideal methods for investigating the structure of dissociative PTSD.

To date, 11 LCA/LPA studies of dissociative PTSD have been published [6]. These studies were all conducted in the USA, Canada or Denmark and, with the exception of one [8], they all identified one latent class (c.f. [9]) which had high levels of both PTSD and dissociation and additionally one or more latent classes that had different levels of PTSD, but relatively low levels of dissociation. The study by Hansen et al. [8], which did not identify a dissociative class, was conducted with a sample of Danish bank employees, all of whom had been victims of bank robbery. The reason for the absence of a dissociative class in their study could possibly be the clearly defined and specific nature of the traumatic event in their sample (compared to other studies, which examined samples exposed to more severe and/or heterogeneous traumatic experiences), although more research is needed to corroborate this assumption. A few of the existing studies which did identify a dissociative class revealed structural differences in PTSD between the dissociative and non-dissociative classes that had comparable levels of PTSD [10–12]. For example, in a sample of 697 US military veterans, Wolf et al. [13] found that the severity of three out of four DSM-5 PTSD symptom clusters (i.e. re-experiencing, negative alterations in cognitions and mood, arousal) was significantly higher in individuals categorized into the high PTSD with dissociation profile than those categorized into the high PTSD only profile. Studies such as these support the structural requirement for the Subtype model of dissociative PTSD by showing that dissociation can affect the severity of PTSD or the endorsement of individual PTSD symptoms.

Another line of research pioneered by Lanius et al. [14, 15] has provided support for the mechanism requirement of the Subtype model of dissociative PTSD. These studies have demonstrated that individuals with dissociative PTSD show differential activation in those brain regions that are implicated in emotion regulation and arousal modulation. More specifically, individuals with PTSD and symptoms of depersonalization or derealization show abnormally high activation in the medial prefrontal cortex and the anterior cingulate cortex and hyper-inhibition of the limbic system [16].

The literature has also supported the meaningfulness requirement for the Subtype model, according to which dissociative and non-dissociative PTSD should differ in their course, effective treatments, co-morbidities or risk factors [5]. Longitudinal studies of PTSD and dissociation have shown that higher baseline levels of dissociation are associated with increased severity of PTSD at a later date [17, 18]. In terms of differential treatment outcomes, there is some evidence suggesting that dissociation may interfere with treatments for PTSD [19, 20], although not all treatment studies reported such an effect [21, 22]. Substantially more evidence for the meaningfulness requirement comes from research examining the co-morbidities and risk factors for dissociative PTSD. These studies aim to determine if any external variables are differentially related to dissociative and non-dissociative PTSD. Significant results have been reported in relation to different psychopathologies [23, 24], social support and emotional coping style [25], childhood adversities [12, 26] and certain demographic variables [13].

### Current study

The primary aim of the current study was to examine the structure and meaningfulness of the dissociative PTSD subtype. An LPA was applied to data from a trauma-exposed sample of university students from Slovakia, where dissociative PTSD has not been examined yet. Additionally, several variables were examined as potential predictors of the latent profiles. These included gender, anxiety and depression, which have been examined in previous studies [10, 24], but also anger, loneliness and distress tolerance, which have not yet been examined in relation to the dissociative PTSD subtype. Anger is a common sequel of trauma exposure [27, 28] and it has been reported to co-occur with both PTSD [29–31] and dissociation [32, 33]. Loneliness has similarly been associated with traumatic exposure [34, 35] and PTSD [36], although to the best of our knowledge it has not yet been examined in relation to dissociation. The final predictor variable examined in this study was distress tolerance, or the ability to withstand negative emotional or other aversive states [37]. Distress tolerance has not been examined in relation to dissociative PTSD before; however, it has been shown to be related to PTSD [38, 39].

The current study also examined whether the DSM-5 definition of the dissociative PTSD subtype should be amended to incorporate a wider range of dissociative experiences (in addition to depersonalization and derealization), a possibility previously highlighted by Dorahy and van der Hart [40] and empirically supported by Műllerová et al. in a similar study [10]. To do so, the LPA utilized in the current study was applied to symptoms of PTSD along with four different symptom clusters of dissociation; depersonalization/derealization, sensory misperceptions, gaps in awareness and memory, and cognitive and behavioural re-experiencing [41]. The current study therefore examined an alternative definition of the dissociative PTSD subtype than the one currently adopted by DSM-5.

It was hypothesized that (1) the LPA will uncover several latent profiles, one of which will be characterized by elevated dissociation symptomatology (i.e. dissociative PTSD profile); (2) compared to the other profiles, the dissociative PTSD profile will score higher on all four dissociative symptom clusters; and (3) gender, depression, anxiety, anger, loneliness and distress tolerance will be differentially related to the dissociative PTSD profile relative to the non-dissociative PTSD profiles.

## Method

### Participants

Participants were recruited from six universities in Slovakia through an email inviting them to participate in a large-scale survey about PTSD. The majority of participants completed the survey online, but pen-and-paper versions were also available at two universities. A total of 2032 students accessed the questionnaire. The survey response rate could not be calculated due to some of the participating universities not updating their mailing lists regularly. The effective sample consisted of 689 trauma-exposed (see the “Measures” section) participants who completed the relevant measures and had less than 30% of missing data on any one relevant measure. The average age of participants in the effective sample was 22.69 years (SD = 5.11) and there were 573 females (83.16%) and 116 (16.84%) males.

### Measures

Traumatic experiences were assessed using the Slovak version of the Life Events Checklist for DSM-5 (LEC-5) [42], which enquires about one’s exposure to 16 potentially traumatic events and one “other very stressful event or experience”. Using a six-point nominal scale (Happened to me, Witnessed it, Learnt about it, Part of my job, Not sure, Does not apply), participants indicated the level of their exposure, with the first four response categories representing a positive endorsement of the experience as per the DSM-5 diagnostic criteria for PTSD [4]. At the end, participants were asked to nominate their worst traumatic event to be used in relation to the assessment of their PTSD symptoms. Of the initial sample of 2032 participants, 1082 completed LEC-5. Participants who did not report any traumatic experiences (*n* = 28), or those who nominated ‘other’ stressful event as most traumatic (*n* = 199), were not included in the analysis. In the latter case, it was not possible to determine whether the experience was traumatic enough to qualify as a DSM-5 PTSD trauma. Participants who did not nominate their worst event, but endorsed the ‘other’ event (*n* = 31), were likewise excluded as their reference event could not be determined.

The Slovak version of the PTSD Checklist for DSM-5 (PCL-5; [43]) was used to assess the 20 DSM-5 symptoms of PTSD. Using a five-point Likert scale ranging from 0 (not at all) to 4 (extremely), participants indicated the extent to which they had been bothered by each symptom in the past month. A cutoff score of 38 was recommended to suggest a probable PTSD diagnosis [44]. Alternatively, following the DSM-5, one or more re-experiencing symptoms, at least one avoidance symptom, two or more negative alterations in cognitions and mood symptoms and at least two arousal symptoms, all rated as 2 = moderately or higher, can be used to indicate a probable diagnosis. Cronbach’s alpha in this study was 0.929.

Dissociation was assessed using the Dissociative Symptoms Scale (DSS; [41]), which was translated into Slovak language using forward- and back-translation by an experienced translator and a doctoral-level psychologist. The authors of the original scale were consulted during the back-translation process. The DSS is a 20-item measure of four domains of dissociation: depersonalization/derealization, sensory misperceptions, gaps in awareness and memory, and cognitive and behavioural re-experiencing. Using a five-point Likert scale ranging from 0 (not at all) to 4 (more than once a day), participants indicated how much each experience happened to them over the past week. Cronbach’s alpha in this study was 0.926 for the whole scale and 0.820, 0.838, 0.775 and 0.772 for the subscales of depersonalization/derealization, sensory misperceptions, gaps in awareness and memory, and cognitive and behavioural re-experiencing, respectively.

Anxiety and depression were assessed using their respective subscales from the Slovak version of the Depression, Anxiety and Stress Scale-21 [45]. Participants indicated the degree to which each statement applied to them in the previous week using a four-point Likert scale ranging from 0 (did not apply to me at all) to 3 (applied to me very much or most of the time). In this study, Cronbach’s alpha was 0.907 for depression and 0.826 for anxiety. The total scores for each subscale were multiplied by 2 as per the guidelines of Lovibond and Lovibond [45].

Anger was assessed with the seven-item version of the Dimensions of Anger Reactions scale [46]. The scale was translated into Slovak language using the procedure outlined above. Participants indicated the degree to which each statement applied to them using a five-point Likert scale ranging from 0 (not at all) to 4 (very much). Cronbach’s alpha was 0.848.

The 20-item Slovak version of the UCLA Loneliness Scale (Version 3; [47]) was used to assess the experiences of loneliness. Participants indicated how often they felt what was described by the statements using a four-point Likert scale ranging from 1 (never) to 4 (always). Cronbach’s alpha in this study was 0.928.

Finally, distress tolerance was assessed using the Slovak version of the Distress Tolerance Scale [48]; a 15-item measure assessing one’s ability to withstand negative psychological states. Responses were recorded using a five-point Likert scale ranging from 1 (strongly agree) to 5 (strongly disagree). Cronbach’s alpha was 0.906.

### Analytic plan

The amount of missing data in the effective sample was 0.096% and Little’s MCAR test suggested that the data was missing completely at random (*χ* ^2^ = 3009.507, *df* = 2921, *p* = 0.124). The expectation maximization algorithm in SPSS 23 was used to impute the missing values prior to the analysis, which was conducted in three steps. Firstly, an LPA was used to categorize individuals into latent profiles based on their responses to the 20 PCL-5 items and their mean scores on the four DSS subscales (please note that the DSM-5 PTSD criteria F, G and H were not assessed in the current study). DSS subscale scores were used instead of symptom scores to facilitate model convergence. This data reduction approach was previously used by Wolf et al. [13] and Műllerová et al. [10] and can be especially useful if the number of LPA indicators is large relative to the sample size. Using the 24 PTSD and dissociation indicators (i.e. 20 PCL-5 symptoms and four DSS subscale mean scores), LPA models with increasing numbers of profiles were estimated in Mplus 7.3 [49] using the robust maximum likelihood estimator. The models were compared using Akaike’s information criterion (AIC), Bayesian information criterion (BIC) and the sample size-adjusted BIC (SSABIC). Lower relative values of these indices point to a better fitting model. The Lo–Mendell–Rubin-adjusted likelihood ratio test (LMRA) was used for direct comparisons of models with different numbers of profiles. Significant *p* values (< 0.05) indicate that a given model fits better than another with one fewer profile. Entropy was used as an indicator of how clearly delineated the profiles were, with values approaching one indicating an accurate classification of participants into latent profiles.

Once the optimal model was selected, analyses of variance (ANOVAs) were used to compare the PTSD and dissociation indicator scores across the profiles. Finally, the three-step approach for conducting multinomial logistic regressions [50] was used to examine the predictors of latent profile membership. With the exception of gender, all predictor variables (depression, anxiety, anger, loneliness, distress tolerance) were continuous and entered into the model as sum scores.

## Results

### Descriptive statistics

Descriptive statistics are presented in Table 1. Based on the PCL-5 cutoff score of 38, 76 participants (11.03%) had probable PTSD. Using the DSM-5 diagnostic algorithm, 122 participants (17.71%) had probable PTSD. The most frequently nominated worst traumatic events were a transportation accident (*n* = 126, 18.29%), natural disaster (*n* = 77, 11.18%), physical assault (*n* = 70, 10.16%), life-threatening illness or injury (*n* = 60, 8.71%) and sudden accidental death (*n* = 59, 8.56%).

**Table 1:** Descriptive statistics of the full sample and each latent profile

Variable	Full sample (*N* = 689)	Non-symptomatic (*n* = 381)	Moderate PTSD (*n* = 210)	High PTSD (*n* = 74)	Dissociative PTSD (*n* = 24)
Probable PTSD^a^ (*n*)					
Yes	122	0	43	56	23
No	567	381	167	18	1
PTSD^b^ (M ± SD)	17.31 ± 15.01	6.82 ± 5.01	23.35 ± 6.44	43.69 ± 10.08	49.50 ± 12.26
Dissociation^b^ (M ± SD)	7.06 ± 9.01	3.03 ± 3.13	9.10 ± 6.86	11.12 ± 6.66	40.63 ± 12.76
Gender (*n*)					
Male	116	77	19	14	6
Female	573	304	191	60	18
Anxiety^b^ (M ± SD)	6.80 ± 7.28	3.60 ± 4.08	8.51 ± 6.62	13.30 ± 9.13	22.50 ± 7.67
Depression^b^ (M ± SD)	7.64 ± 9.08	3.80 ± 4.72	8.72 ± 8.13	17.35 ± 10.51	29.33 ± 9.79
Anger^b^ (M ± SD)	6.96 ± 5.64	5.15 ± 4.21	7.66 ± 5.06	10.88 ± 7.38	17.33 ± 5.70
Loneliness^b^ (M ± SD)	40.05 ± 10.90	36.18 ± 8.81	42.23 ± 10.17	48.43 ± 11.60	56.58 ± 10.06
Distress tolerance^b^ (M ± SD)	51.10 ± 11.90	54.81 ± 11.23	48.46 ± 10.21	43.49 ± 12.33	38.88 ± 9.57

^a^ Probable PTSD based on the DSM-5 diagnostic algorithm

^b^ Total score

### Latent profiles

Table 2 shows the fit statistics of the estimated models. The five-profile model yielded an unreliable solution due to a non-positive definite first-order derivative product matrix, and will therefore not be discussed here. The values of the AIC, BIC and SSABIC indices were the lowest for the four-profile model, although the LMRA test favoured the two-profile model. All models had good entropy values (> 0.9) and therefore the four-profile model was selected as best fitting.

**Table 2:** Fit indices of the estimated latent profile models

Model	AIC	BIC	SSABIC	LMRA *p* value	Entropy
One profile	46,761.022	46,978.714	46,826.306	–	–
Two profiles	41,941.364	42,272.436	42,040.650	0.0001	0.971
Three profiles	40,514.021	40,958.475	40,647.310	0.3071	0.961
Four profiles	39,647.783	40,205.618	39,815.074	0.2246	0.940

*AIC* Akaike’s information criterion, *BIC* Bayesian information criterion, *LMRA* Lo–Mendell–Rubin-adjusted likelihood ratio test, *SSABIC* sample size-adjusted BIC

**Fig. 1:**
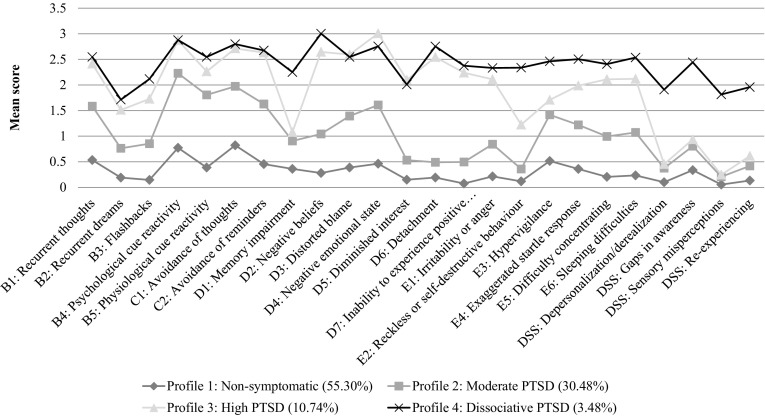
Four-class profile plot of the PTSD symptom scores and the dissociation subscale scores

Figure 1 shows the profile plot of the optimal model. Based on the most likely class membership, Profile 1 consisted of 55.30% (*n* = 381) of the sample and was named non-symptomatic. These individuals scored low on all PTSD and dissociation indicators. Profile 2 consisted of 30.48% (*n* = 210) of the sample and was named moderate PTSD. Relative to the non-symptomatic profile, these individuals had elevated PTSD symptomatology and slightly elevated dissociation symptomatology. Profile 3, consisting of 10.74% (*n* = 74) of the sample, was named high PTSD. These individuals had, on average, higher PTSD symptom scores than those in the non-symptomatic and moderate PTSD profiles, but their scores on dissociation indicators remained relatively low. Finally, Profile 4 was the smallest subgroup, consisting of 3.48% (*n* = 24) of the sample. With the exception of a few PTSD symptoms, it was characterized by PTSD symptomatology comparable to that in the high PTSD profile, but it had elevated scores on all four dissociation indicators. Profile 4 was therefore named dissociative PTSD.

### PTSD and dissociation as a function of latent profile membership

The ANOVAs examining the differences in PTSD and dissociation symptomatology as a function of latent profile membership were all significant (*p*_s_ < 0.001), even after applying the Bonferroni correction to account for multiple comparisons. The Games-Howell post hoc tests, which are suitable for examining differences between samples of unequal sizes and with unequal variances, showed that all PTSD and dissociation scores in the non-symptomatic profile were significantly lower (*p*_s_ < 0.001) than those in the other three profiles. Cohen’s *d* effect sizes for PTSD ranged from 0.440 to 2.909 and for dissociation from 0.721 to 3.082. When comparing the moderate PTSD with the high PTSD profile, the latter one had significantly higher scores on all PTSD symptoms (*p*_s_ ≤ 0.001, Cohen’s *d* = 0.534 to 2.297) except for B5, D1 and E3 (see Fig. 1 for symptom descriptions). The high PTSD profile also had significantly higher scores on the dissociative subscale of cognitive and behavioural re-experiencing (*p* = 0.033, Cohen’s *d* = 0.398), but none of the other dissociative subscales. Comparing the moderate PTSD profile with the dissociative PTSD profile, the latter scored significantly higher on all PTSD symptoms (*p*_s_ < 0.05, Cohen’s *d* = 0.637 to 1.990), except for B4 and B5, and it also scored higher on all dissociation indicators (*p*_s_ < 0.001, Cohen’s *d* = 2.017 to 2.687). Finally, the high PTSD and the dissociative PTSD profiles did not differ significantly on any PTSD symptoms, except for D1 (*p* = 0.005, Cohen’s *d* = 0.866) and E2 (*p* = 0.008, Cohen’s *d* = 0.821), where the dissociative PTSD profile scored significantly higher. The dissociative PTSD profile also scored significantly higher than the high PTSD profile on all four dissociation indicators (*p*_s_ < 0.001). The effect sizes ranged from 1.765 for cognitive and behavioural re-experiencing to 2.623 for Sensory misperceptions.

### Predictors of latent profiles

The results of the multinomial logistic regressions are presented in Table 3. Males were significantly less likely to be in the moderate PTSD profile than in the non-symptomatic, high PTSD or dissociative PTSD profiles. There was no differentiation based on gender between the high PTSD and dissociative PTSD profiles. In relation to anxiety, for every one point increase on the anxiety measure, individuals were significantly more likely to be in the moderate PTSD, the high PTSD or the dissociative PTSD profiles than the non-symptomatic profile, and significantly more likely to be in the dissociative PTSD profile than the moderate or high PTSD profiles. Depression emerged as a significant predictor of the non-dissociative PTSD profiles, such that for every one point increase in depression, individuals were significantly more likely to be in the higher severity PTSD profiles. There was no differentiation based on depression between the high PTSD and the dissociative PTSD profiles. Anger was a significant predictor of the dissociative PTSD profile relative to all other profiles, such that higher levels of anger predicted membership in the dissociative PTSD profile. Interestingly, there was no differentiation based on anger between the non-dissociative PTSD profiles. Loneliness was a significant predictor of the moderate PTSD and high PTSD profiles relative to the non-symptomatic profile and distress tolerance did not predict membership in any of the latent profiles.

**Table 3:** Odds ratios and 95% confidence intervals for predictors of the latent profiles

Predictors	Non-symptomatic^a^ vs. moderate PTSD	Non-symptomatic^a^ vs. high PTSD	Non-symptomatic^a^ vs. dissociative PTSD	Moderate PTSD^a^ vs. high PTSD	Moderate PTSD^a^ vs. dissociative PTSD	High PTSD^a^ vs. dissociative PTSD
Male sex^b^	0.324 (0.162–0.650)**	0.761 (0.319–1.814)	1.826 (0.478–6.977)	2.347 (1.004–5.483)*	5.629 (1.526–20.767)**	2.399 (0.695–8.279)
Anxiety	1.125 (1.069–1.184)***	1.138 (1.069–1.211)***	1.280 (1.152–1.423)***	1.011 (0.963–1.062)	1.138 (1.034–1.252)**	1.125 (1.022–1.239)*
Depression	1.062 (1.013–1.113)**	1.135 (1.073–1.202)***	1.195 (1.094–1.305)***	1.069 (1.026–1.114)**	1.125 (1.038–1.219)**	1.052 (0.975–1.136)
Anger	1.024 (0.975–1.076)	1.060 (0.986–1.139)	1.206 (1.080–1.345)**	1.035 (0.974–1.099)	1.177 (1.063–1.303)**	1.139 (1.035–1.254)**
Loneliness	1.038 (1.014–1.062)**	1.047 (1.007–1.089)*	1.036 (0.967–1.109)	1.008 (0.973–1.044)	0.998 (0.932–1.069)	0.990 (0.928–1.056)
Distress tol.	0.983 (0.962–1.005)	0.969 (0.937–1.001)	1.001 (0.942–1.064)	0.985 (0.957–1.015)	1.018 (0.960–1.080)	1.034 (0.976–1.094)

* <.05; ** <.01; *** <.001

^a^ Reference profile

^b^ ‘Female’ was the reference category

## Discussion

The current study examined the structure and meaningfulness of the dissociative PTSD subtype, using an extended set of dissociative symptoms. The results of the LPA revealed four latent profiles differing in the severity of PTSD and dissociation symptomatology: non-symptomatic, moderate PTSD, high PTSD and dissociative PTSD. The last two profiles differed primarily in the severity of dissociation indicators, which was higher in the dissociative PTSD profile, thus supporting hypothesis 1. Additionally, the dissociative PTSD profile had elevated scores on all dissociation indicators (not just depersonalization and derealization), which supports hypothesis 2. Finally, hypothesis 3 was partly supported, as anxiety and anger, but none of the other variables, differentially predicted membership in the dissociative PTSD profile relative to the high PTSD profile.

In line with previous studies [9–13, 23–26, 51], the current results support the existence of the dissociative PTSD subtype, as only a small proportion of individuals with elevated PTSD symptomatology also reported elevated dissociative symptoms. The results also contribute to the notion that dissociative PTSD is not a culture-specific construct limited to the USA, Canada and Denmark, as the current study identified its existence in a sample from Slovakia, where this construct had not been examined before. Stein et al. [52] (non-LCA/LPA study) examined the existence of the dissociative subtype in the World Mental Health Survey conducted across 16 countries and found that 14.4% of individuals with a 12-month diagnosis of PTSD reported symptoms of dissociation. The current study adds to the findings of Stein et al. who did not include Slovakia in their investigation, although they did include two Eastern European countries; Bulgaria and Romania.

The results further showed that the high PTSD and the dissociative PTSD profiles differed significantly not only in the severity of dissociative symptoms, but also in the severity of two core PTSD symptoms; memory impairment (D1) and reckless or self-destructive behaviour (E2). These findings support the structural requirement for the subtype hypothesis of dissociative PTSD [5], because dissociation in the current sample essentially changed the structure of PTSD. The different severity of the memory impairment symptom in the two latent profiles is not surprising. Indeed, it has previously been suggested that psychogenic amnesia is a symptom of dissociation [40, 53, 54]. Stein et al. [52] reported that in their sample of 25,018 respondents, amnesia and flashbacks were the only PTSD symptoms that were significantly associated with depersonalization and derealization. Interestingly, there was no differentiation based on flashbacks between the two profiles in the current study.

In relation to symptom E2 (reckless or self-destructive behaviour), which was only recently added to the DSM PTSD criteria [4], Frewen et al. [9] found that individuals in their severe dissociative PTSD profile, as well as those in the moderate dissociative PTSD profile endorsed this symptom with greater severity than individuals in the severe non-dissociative PTSD profile. Taken together, the findings of the current study and those of Frewen et al. seem to suggest that the PTSD symptom E2 could be specific to the dissociative PTSD subtype.

Further support for memory impairment and reckless or self-destructive behaviour symptoms as poor indicators of (non-dissociative) PTSD comes from the PTSD confirmatory factor analytic literature, where substantially low factor loadings have been reported for the two symptoms [55–57]. Memory impairment and reckless or self-destructive behaviour symptoms may therefore potentially be better indicators of the dissociative PTSD subtype than the non-dissociative PTSD.

The current study also demonstrated that the dissociative PTSD profile could be identified on the basis of an extended set of dissociative symptoms. The DSS subscales of depersonalization/derealization, sensory misperceptions, gaps in awareness and memory, and cognitive and behavioural re-experiencing all differentiated between the high PTSD and the dissociative PTSD profiles. The DSM-5 limits the diagnosis of dissociative PTSD to symptoms of depersonalization and derealization; however, the current study showed that such a restriction may be inaccurate. This conclusion is supported by the existing literature, according to which a wide range of dissociative symptoms may follow traumatic exposure [58]. So far, there have only been two LPA studies that examined an extended set of dissociative symptoms in PTSD [10, 13]. The study by Műllerová et al. [10] used the same measures of PTSD and dissociation as the current study and it similarly found that the dissociative PTSD profile could be characterized by elevated scores on all DSS subscales, with Cohen’s *d* effect size being largest for the subscale of cognitive and behavioural re-experiencing. In the current study, the largest effect size was found for sensory misperceptions. These findings will need further replications; however, both studies suggest that the symptoms of depersonalization and derealization should not be given precedence in the diagnosis of the dissociative PTSD subtype.

The current study also examined the meaningfulness requirement for the subtype hypothesis of dissociative PTSD by looking at variables that could potentially predict membership in the dissociative PTSD profile relative to the high PTSD profile. Contrary to our predictions, there was no difference based on depression, gender, loneliness and distress tolerance between the two profiles. To date, loneliness and distress tolerance have not been examined as predictors of dissociative PTSD and the results regarding depression and gender have been mixed so far [10, 24, 26]. Further research employing these variables is needed to establish their predictive value for dissociative PTSD. Anxiety and anger have, however, emerged as significant predictors of the dissociative PTSD profile in the current study. Specifically, individuals with high levels of PTSD who reported high levels of anxiety were more likely to be in the dissociative PTSD profile than the high PTSD profile. Such results have been reported in several of the previous studies [10, 23, 24], suggesting that anxiety may be an important risk factor for dissociative PTSD. Considering the fact that the high and dissociative PTSD profiles in the current sample had comparable levels of PTSD, it can be suggested that anxiety contributes to the expression of dissociative symptoms in the dissociative PTSD subtype, above and beyond its relationship with PTSD. This finding supports the previously suggested notion that dissociative PTSD is associated with greater co-morbidity [26]. It is also in line with the proposition that dissociation can serve as a defence mechanism [59] against adverse emotional states, such as anxiety. However, considering the fact that the relationship between anxiety and the dissociative PTSD subtype in previous studies has not been consistent [6], further research is needed to disentangle this relationship.

Our results also showed that higher levels of anger were significantly associated with the dissociative PTSD profile membership relative to all non-dissociative PTSD profiles. However, there was no differentiation based on anger between any of the non-dissociative profiles. These findings provide support for the specificity of anger for the dissociative PTSD subtype and concur with previous studies which have found a significant association between anger and dissociation even after controlling for PTSD severity [32, 33]. Feeny et al. [32] argued that anger and dissociation are related, because they are complementary forms of emotional disengagement from traumatic memories. Both methods prevent the processing of traumatic memories, thus hindering recovery. More research is, however, needed to better understand why anger would be a differential predictor of dissociative PTSD relative to non-dissociative PTSD.

### Limitations

Certain limitations of the current study need to be acknowledged. First, the DSS items were not queried in the context of participants’ worst trauma, which could potentially mean that they did not result from the traumatic event. Second, the indicators for the LPA were individual PTSD symptoms, whereas for dissociation we used mean subscale scores. This prevented us from determining the importance of the individual dissociative symptoms for the dissociative PTSD subtype. Nevertheless, considering the relatively small sample size, this approach enabled us to use a full validated measure of dissociation. Third, all data was collected using self-report measures rather than the gold-standard clinical interviews. In relation to the PCL-5, this measure can only be used to indicate a probable PTSD diagnosis. However, in previous studies [60, 61], it showed good psychometric properties and in the current study it had good internal consistency. Nevertheless, the study will need to be replicated using structured clinical interviews. Future studies could utilize samples consisting of only PTSD-diagnosed individuals to see if similar latent profiles emerge. Finally, the use of a predominantly female non-clinical student sample limits the generalizations to male and/or clinical samples. However, having said that, two of the previous LPA studies replicated their findings in a subsample of their participants who met the PTSD criteria [12, 24], thus suggesting some degree of generalizability to clinical samples. The effects of gender on dissociative PTSD are not yet fully understood and therefore generalizations from this study should only be made with caution, as 83.16% of participants were female.

### Implications

Despite these limitations, the results of this study have important implications. Firstly, since all of the DSS subscale scores were significantly elevated in the dissociative PTSD profile, limiting the dissociative PTSD subtype to symptoms of depersonalization and derealization may be inaccurate. Some individuals may display dissociative symptoms other than depersonalization and derealization and experience similar functional impairment or treatment non-response as those with depersonalization and derealization. Further research in this area is needed, but if the results are replicated in future studies, a revision to the DSM-5 diagnostic criteria for PTSD may be necessary. Secondly, the PTSD symptoms D1 and E2 appear to be better indicators of the dissociative PTSD subtype than the non-dissociative PTSD, as they were more highly endorsed by individuals in the dissociative PTSD profile than all others. If replicated in future studies, the designation of these symptoms as core symptoms of PTSD may need to be reconsidered. Thirdly, anger was found to be a specific predictor of dissociative PTSD, which may have implications for treatments of individuals with the dissociative PTSD subtype, as there is some evidence suggesting that anger may interfere with exposure treatments for PTSD [62–64]. There is also some evidence suggesting that dissociation may interfere with treatments [19, 20]. Future studies should therefore examine the effects of different types of treatments on dissociative PTSD as well as different techniques to counteract dissociative responding in therapy [65], both in individuals who do and those who do not display anger. Finally, the identification of a dissociative subtype in a European sample suggests that the omission of this diagnostic construct from the proposed 11th edition of the International Classification of Diseases may be a mistake.

## Conclusion

The current study used LPA to investigate the Subtype hypothesis of dissociative PTSD in a sample of university students from Slovakia. Strong support was found for both the structural and meaningfulness requirement of the Subtype hypothesis. In terms of the definitional requirement, the results suggest that the set of dissociative symptoms characterizing the DSM-5 dissociative PTSD subtype could be extended to include alternative dissociative experiences. Further studies with different populations and examining a variety of dissociative symptoms are warranted.
